# Healthcare Resource Utilisation in Patients with Upper Tract Urothelial Carcinoma

**DOI:** 10.3390/healthcare14121729

**Published:** 2026-06-16

**Authors:** Sanjib Saha, Tove Sundström, Johannes Bobjer, Fredrik Liedberg, Elin Ståhl

**Affiliations:** 1 Health Economics Unit, Department of Clinical Science (Malmö), Lund University, Forum Medicum, Sölvegatan 19, SE-223 62 Lund, Sweden; 2 Department of Urology, Skåne University Hospital, Department of Translational Medicine, Lund University, SE-205 02 Malmö, Sweden; tove.sundstrom@skane.se (T.S.); johannes.bobjer@med.lu.se (J.B.); fredrik.liedberg@med.lu.se (F.L.); elin.stahl@med.lu.se (E.S.)

**Keywords:** cost of illness, direct cost, healthcare resource utilisation, radical nephroureterectomy, endoscopic treatment, kidney-sparing surgery, segmental ureterectomy, upper tract urothelial carcinoma

## Abstract

**Background:** Upper tract urothelial carcinoma (UTUC) is rare, and contemporary data on real-world healthcare resource utilisation and costs are limited. The objective of this study is to describe long-term healthcare resource utilisation among patients with upper tract urothelial carcinoma (UTUC) and to identify clinical and treatment-related drivers of costs. **Methods:** We conducted a retrospective, population-based cohort study including all patients diagnosed with UTUC between 2019 and 2023 in Region Skåne, Sweden. Patients were identified through the Swedish National Register for Urinary Bladder Cancer (SNRUBC) and linked to regional healthcare databases covering primary, secondary, and tertiary care. The primary outcome was annual direct healthcare cost per patient, derived from Diagnosis-Related Group (DRG) cost data and expressed in 2023 international dollars (Int$). Secondary outcomes were cost patterns and predictors stratified by treatment modality: robot-assisted nephroureterectomy (RANU), open nephroureterectomy (ONU), segmental ureterectomy (SU), and endourological treatment (ET). **Results:** Among 278 included patients, most were older adults and/or with substantial comorbidity, and over half underwent radical nephroureterectomy. The adjusted mean annual cost was Int$36,870 in 2019, decreasing to Int$30,004 in 2023. In the subgroup treated with ONU, systemic treatment was associated with a higher adjusted cost ratio and in the subgroup operated with SU, female sex was associated with a higher adjusted cost ratio. Comorbidity was a cost driver in the ET subgroup. **Conclusions:** UTUC care in this Swedish region has become less resource-intensive over a short period. These results can provide a basis for planning UTUC services and highlight targets for cost-conscious, patient-centred optimisation of care.

## 1. Introduction

Upper tract urothelial carcinoma (UTUC) is a rare urological malignancy originating from the urothelial lining of the renal pelvis and ureter. Although it shares histological similarities with bladder cancer, its epidemiology, clinical management, and treatment are very different [1]. UTUC constitutes about 5–10% of all urothelial carcinomas and the incidence varies according to age, certain risk-occupations as well as geographical regions [2]. The age-standardised incidence of UTUC in Sweden is two to three cases per 100,000 individuals annually [3]. The rarity of the condition, coupled with diagnostic challenges, and the broad range of treatment options (supported primarily by low-level evidence) make disease management challenging [4]. Thus, patients with UTUC may be subjected to radical nephroureterectomy (RNU) or kidney-sparing surgery (KSS) with either segmental ureterectomy (SU) or endourological treatment (ET) [5].

The disease burden extends beyond direct medical costs and includes repeated imaging, inpatient admissions, and long-term surveillance due to a high recurrence rate, especially recurrences in the bladder, i.e., intravesical, as well as ipsilateral upper tract recurrence after KSS. Despite its clinical significance, research on healthcare resource utilisation (HCRU) in UTUC remains limited compared to other urological cancers. The existing literature is limited by mainly consisting of low-level evidence, short follow-up periods, insufficient clinical granularity and focus on low-grade UTUC, as identified in a prior systematic review [6]. Thacker et al.’s systematic review exemplifies these constraints, noting that only 15 studies met inclusion criteria, that many cost estimates depended on a single 2009 scenario analysis, and that downstream events such as dialysis, chronic kidney disease progression, recurrences, and long-term complications were poorly captured [6].

To assess contemporary, population-based real-world HCRU in UTUC, we used the Swedish National Register for Urinary Bladder Cancer (SNRUBC) that registers all patients with UTUC in Sweden since 2015 with high coverage [3]. The primary objective was to estimate healthcare resource utilisation among patients with UTUC in Region Skåne, Sweden. The secondary objective was to perform subgroup analyses according to the primary treatment modality, including RNU (with either robot-assisted nephroureterectomy (RANU) or open nephroureterectomy (ONU)) and KSS (with SU or ET). Detailed knowledge about HCRU will facilitate the identification of high-cost care components, variation across disease stages and treatment modalities, and potential areas for interventions to reduce unnecessary expenditures while improving patient outcomes.

## 2. Materials and Methods

### 2.1. Study Population and Variables

All patients diagnosed with primary UTUC within four years (2019–2023) in Region Skåne, Sweden (population size 1.42 million), were retrieved from the SNRUBC. Using the unique Swedish personal identity number, we linked all inpatient and specialist outpatient contacts recorded in the Skåne Healthcare Register to each study participant. Data from the SNRUBC including date of diagnosis, age, smoking status (never, former or current smoker), clinical tumour stage (according to TNM 2017), multidisciplinary tumour board discussion (MDT), primary treatment and treatment intent were linked to Region Skåne’s healthcare databases (Region Skåne VårdDatabaser, RSVD) [7]. Additional data on body mass index (BMI), comorbidity (ASA-score), functional single kidney, history of bladder cancer, diagnostic procedures, tumour size, tumour site, local invasion on computed tomography (CT), tumour multiplicity including risk stratification according to EAU risk categories [1], pathological tumour stage groups (Ta/Tx and G1, Ta/Tx and G2, Ta/carcinoma in situ (CIS)/T1/Tx and G3, and T2–T4 (any grade); WHO grade 1999) and nodal stage in the lymphadenectomy specimens were retrieved by chart review. BMI was categorised as normal weight (18.5–24.9 kg/m^2^), overweight (25.0–29.9 kg/m^2^), or obese (≥30.0 kg/m^2^) according to WHO criteria [8]. Treatment types were categorised as follows: RANU, ONU, SU (either robot-assisted distal ureterectomy (RADU) or open distal ureterectomy (ODU)), endourology (ET), palliative systemic treatment, and best supportive care only.

From RSVD, data was extracted for inpatient hospitalisations, outpatient consultations, primary care visits, diagnostic procedures, surgical interventions, systemic and intravesical therapies, and radiotherapy. Corresponding cost information was derived from the Swedish Diagnosis-Related Group (DRG) cost register within RSVD [9].

**Figure 1:**
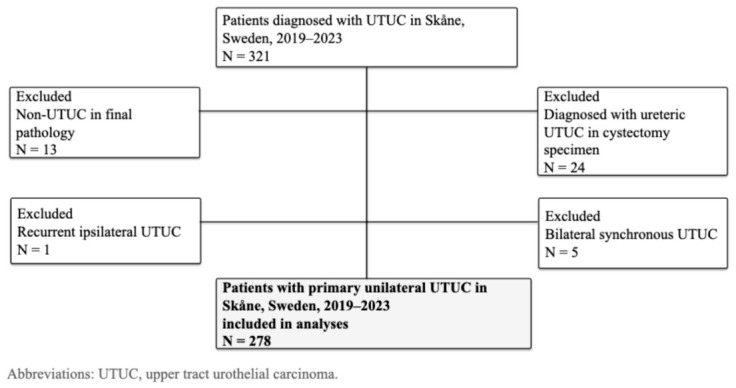
CONSORT flow diagram describing and defining the investigated population-based cohort.

The analysis covers the period from first diagnosis through follow-up, up to the year 2023. Among the 321 identified patients, exclusions comprised 24 patients diagnosed with synchronous ureteric UTUC in cystectomy specimens (distal ureteric tumour in the cystectomy specimen, usually carcinoma in situ), five with bilateral synchronous UTUC (different cost and clinical consideration due to bilaterality), 13 with a non-UTUC diagnosis, and one with recurrent ipsilateral UTUC (Figure 1).

### 2.2. Outcomes

The primary outcome was direct annual healthcare cost per UTUC patient. This encompassed inpatient, outpatient, and primary care services delivered within both the public and private sectors in Region Skåne. Episode-level costs were assigned using DRG codes, which cluster diagnoses with similar resource requirements. For each healthcare unit, the annual total cost was divided by the total number of DRG points generated to derive a cost per DRG point, in accordance with standard regional accounting practice.

Annual direct healthcare costs were calculated as the sum of DRG-based tariffs for all relevant inpatient and specialist outpatient contacts within each calendar year (2019–2023)—including diagnostic procedures, surgical and oncological treatments, follow-up visits, and management of recurrences—such that each observation represents the total cost incurred by a patient in that year, irrespective of time since diagnosis, thereby allowing trends in costs over calendar time to be described from a health-system perspective. All costs were adjusted to 2023 prices using the consumer price index (CPI) [10] and converted to 2023 international dollars using purchasing power parity (PPP) [11].

### 2.3. Subgroup Analyses

Four subgroups of patients treated with robot-assisted nephroureterectomy (RANU), open nephroureterectomy (ONU), segmental ureterectomy (SU) and endourologic treatment (ET) were investigated separately to explore the costs by treatment type. These subgroup analyses were based on relatively small sample sizes and were therefore considered exploratory and only hypothesis-generating.

### 2.4. Statistical Analyses

Descriptive statistics summarise patient demographics, disease characteristics and number of outpatient contacts and inpatient days, including Pearson’s chi-square test for association between treatment categories and calendar year. Univariable and multivariable regression models identify sociodemographic, clinical, and treatment-related factors associated with higher healthcare utilisation and costs. We modelled annual healthcare costs by calendar year (2019–2023) using generalised linear models with a gamma family and log link, which are appropriate for positively skewed cost data. For each calendar year, the outcome was defined as total healthcare costs among individuals alive in that year. To account for differential probabilities of being alive and observed for a specific calendar year (Supplementary Table S1), we applied year-specific inverse probability weights in all cost models.

We first estimated unadjusted mean annual costs using a pooled model including only indicator variables for calendar year. We then fitted multivariable models adjusting for baseline, clinical and treatment characteristics. In all analyses, we treated each person-year as an observation and used cluster-robust standard errors at the individual level to allow for within-person correlation over time, yielding population-averaged estimates of mean annual costs by calendar year and covariate patterns.

Covariates included in the univariable and multivariable analyses were not identical across all models. Selection of covariates for the overall UTUC cohort and for the subgroup analyses of RANU, ONU, SU, and ET was guided by the researchers’ clinical expertise and judgement. Thus, selection of covariates in the subgroup analysis of ET was restricted by omitting clinical tumour stage, clinical nodal stage and systemic treatment because ET is not indicated in higher tumour stages, presence of nodal metastasis, and when systemic treatments are considered.

For covariates with missing values (e.g., smoking status, tumour size), we did not exclude patients or perform multiple imputation. Instead, we created an explicit “missing” category and included this as a separate level in the regression models. This missing-indicator approach allowed us to retain all individuals in the analyses while adjusting for potential differences between those with recorded and unrecorded values.

Because we conducted several subgroup analyses with multiple covariates, we controlled the family-wise type I error rate using Bonferroni correction [12]. For each model, we treated all non-reference covariates as one family of hypotheses and obtained Bonferroni-adjusted *p*-values by multiplying the original *p*-values by the number of tests in that model, truncating any adjusted values at 1. Associations were considered statistically significant only if the corresponding Bonferroni-adjusted *p*-value was <0.05. We used STATA version 19 for all the statistical analyses.

## 3. Results

### 3.1. Patient Characteristics

A total of 278 patients with UTUC were included (Table 1). Most patients were older adults, with 88% aged 65 years or older and 22% aged 81 years or above. Men accounted for 59% of the cohort, and the majority of patients had comorbidities, with 157 (56%) categorised as ASA score 3 or 4. Smoking was also common: 82 (29%) were former smokers, 71 (26%) current smokers, and 23% had missing smoking information. Most patients (n = 156 (56%)) had a renal pelvic tumour (or, in cases with multiple tumours, the largest tumour location).

### 3.2. Inpatients Days, Outpatient Visits and Treatment Types

Mean annual inpatient hospital days per patient-year ranged from approximately 9 to 24 days across diagnosis cohorts and calendar years, with overlapping standard deviations indicating substantial within-group variability (Supplementary Table S2). Mean annual outpatient contacts per patient-year ranged from approximately 12 to 26, remained relatively stable over time, and showed overlapping variability across diagnosis cohorts (Supplementary Table S3). The distribution of treatment types did not differ significantly by calendar year (Pearson’s chi-square *p* = 0.11), indicating no shift in treatment patterns over the study period (Supplementary Table S4).

### 3.3. Overall Cost Trends

**Figure 2:**
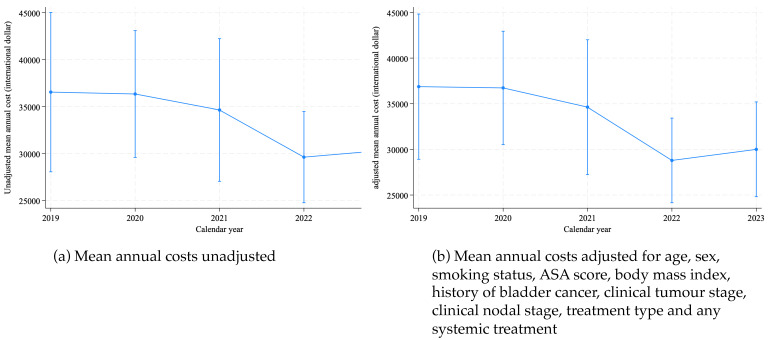
Mean annual costs for patients with upper track urothelial carcinoma in Skåne, Sweden (2019–2023). Each calendar year with (**a**) unadjusted and (**b**) adjusted genaralised linear model. Adjusted for age, sex, smoking status, ASA score, body mass index, history of bladder cancer, clinical tumour stage, clinical nodal stage, treatment type and any systemic treatment.

Annual direct healthcare costs varied over the study period but remained high throughout. In the adjusted gamma log-link model, the predicted mean annual cost was 36,870 international dollars (95% CI 28,915–44,825) in 2019 and 30,004 (95% CI 24,817–35,191) in 2023, with no statistically significant differences between calendar years (all *p* > 0.05) (Figure 2 and Supplementary Table S5). The unadjusted GLM showed a similar pattern, with predicted mean costs ranging from 29,614 to 36,536 international dollars across years and overlapping confidence intervals (Figure 2 and Supplementary Table S5). Costs tended to decline over follow-up for the cohort diagnosed in 2019 but showed no consistent temporal trend overall (Supplementary Table S6).

### 3.4. Multivariable Analysis

In the multivariable analysis, only systemic treatment was associated with higher costs in the adjusted model (adjusted cost ratio 1.52, 95% CI 1.13–2.04; *p* = 0.005), but it was not significant after Bonferroni correction (Table 2).

### 3.5. Subgroup Analyses

The subgroup analyses showed a few pattern-specific findings. Among patients treated with RANU, higher ASA score carried a stronger cost burden, with ASA 3–4 associated with increased costs compared with ASA 1 (adjusted cost ratio 2.01, 95% CI 1.16–3.50; *p* = 0.013), and a history of bladder cancer was also linked to higher costs (adjusted cost ratio 1.83, 95% CI 1.09–3.04; *p* = 0.021) (Supplemental Table S7). Among patients treated with ONU, systemic treatment had higher adjusted cost ratio (2.38, 95% CI 1.56–3.62; *p* < 0.001) also after Bonferroni correction (Supplemental Table S8). For patients treated with SU, female sex was linked to higher costs with an adjusted cost ratio of 1.82 (95% CI 1.30–2.55; *p* < 0.001, which remained significant after Bonferroni correction (Supplemental Table S9). Among patients treated with ET, higher ASA score was associated with higher costs, with adjusted cost ratios for ASA 2 (3.22, 95% CI 1.55–6.71; *p* = 0.002) and ASA 3–4 (5.46, 95% CI 3.18–9.37; *p* < 0.001), respectively, with significance remaining also after Bonferroni correction (Supplemental Table S10). The treatment-specific costs are presented in Supplementary Tables S11–S14.

## 4. Discussion

### 4.1. Decreasing Healthcare Costs over Time

Mean annual direct healthcare cost per patient diagnosed with UTUC between 2019 and 2023 decreased from 36,870 to 30,004 international dollars after adjustment. This downward trajectory was consistent across all calendar years and remained statistically significant regardless of covariate adjustment, suggesting that the decrease reflects a genuine and sustained shift in the resource intensity of UTUC management rather than a compositional change in the patient population. These findings contrast with prior earlier work from the United States, where Thacker et al. documented a decade-long rise in the utilisation of costly minimally invasive nephroureterectomy and noted that direct surgical costs were increasing as robot-assisted procedures displaced open surgery [6]. Similarly, Tinay et al. reported that RANU carried the highest direct hospital costs among all RNU approaches in a large US database, driven primarily by supply and operating room costs [13], where on the other hand subgroup analyses in patients operated with RANU and ONU in the current study showed a decrease in adjusted direct healthcare costs per patient during the study period for both methods. In the current study, all RANUs were performed in one high-volume tertial referral centre with only 1% high-grade complications [14]. Prior studies have shown that surgery at high-volume centres are associated with lower cost [15]. There is also some support for improved survival after RANU/ONU performed in high-volume hospitals compared to low-volume hospitals [16], and thus the high-volume context in the current study might have affected the development of costs.

### 4.2. The Role of Age and Comorbidity

Patient age did not seem to influence adjusted healthcare costs, except in the subgroup treated with SU where individuals with an age of 81 years or older incurred higher annual costs than those under 65 (adjusted cost ratio 1.97, 95% CI 1.23–3.15; *p* = 0.005). In the subgroup of patients treated with ET, an ASA score of 3–4 was also associated with elevated costs, which is not unexpected. UTUC predominantly affects older patients, and a large proportion carry significant comorbidity burdens including cardiovascular disease, diabetes, and chronic kidney disease [17]. In older and comorbid patients not eligible for nephroureterectomy, attempts with ET instead of radical surgery might have contributed to this finding through such selection mechanisms. Each of these consequences generates downstream healthcare contacts such as longer hospital stays, readmissions, and extended follow-up which, in turn, accumulate into higher overall costs.

### 4.3. Sex Differences in Healthcare Costs in the SU Subgroup

Female sex was not associated with higher adjusted annual costs in the overall cohort, but was in the SU subgroup (adjusted cost ratio 1.82; 95% CI 1.30–2.55; *p* < 0.001). Sex-based differences in UTUC are well documented: women tend to present with more advanced tumour stages and have higher cancer-specific mortality in advanced disease compared with men [18]. This later-stage presentation is likely to require more intensive treatment such as perioperative lymphadenectomy and perioperative systemic therapy, extended surveillance, and more frequent retreatment, all of which translate into higher registered healthcare costs over time.

### 4.4. Treatment Modality and Costs

One reassuring finding was that, after adjustment, the treatment modality, whether RANU, ONU, SU, ET, or conservative management, was not independently associated with significantly different healthcare costs. This aligns with the hypothesis that the patient’s underlying clinical profile, comorbidity burden, and disease characteristics rather than the surgical approach itself are the primary determinants of overall cost. Comparative studies demonstrate that kidney-sparing management yields significant cost savings compared to RNU during initial episodes of care [19]. This is primarily due to the avoidance of the substantial upfront procedural costs and the long-term consequences of kidney loss, including chronic kidney disease and end-stage renal disease.

### 4.5. Strength and Limitations

Several limitations should be acknowledged. The study was conducted in a single Swedish region, and while Region Skåne is large and demographically heterogeneous, the findings may not fully generalise to the whole of Sweden or to other healthcare systems. The study period of 2019–2023 coincides in part with the COVID-19 pandemic, which disrupted cancer care pathways and may have compressed or displaced healthcare contacts in 2020 and 2021, potentially affecting cost estimates in those years.

Another limitation is that we did not break down total costs into specific components (diagnostics, surveillance, treatment of recurrences); our focus was on overall annual healthcare costs per patient. Future studies using the same registers could provide more granular cost estimates by clinical phase or resource category. A further limitation is that some key covariates, notably smoking status and tumour size, had substantial missingness. We treated missing values as a separate category rather than imputing them, because clinical experience and data patterns suggest that missingness is unlikely to be at random, especially for smoking status. While this approach avoids loss of sample size, we cannot exclude residual confounding or bias associated with informative missingness. Still, the subgroup analyses, particularly for SU and ET, were limited by small sample sizes, which increase the risk of overfitting in adjusted models and yield unstable, imprecise estimates; consequently, subgroup findings are only hypothesis-generating. Finally, indirect costs such as productivity loss, informal caregiving, and patient out-of-pocket expenditure were not captured, meaning that the true societal burden of UTUC is likely to be higher than the direct costs reported here.

This study has several notable strengths. The use of Region Skåne’s comprehensive healthcare database enabled near-complete capture of inpatient, outpatient, and primary care contacts across public and private sectors, providing a more complete representation of healthcare utilisation than is achievable with local hospital registries or national inpatient databases. Not all regions in Sweden collect and register primary care visits. The DRG-based cost calculation reflects actual resource weights rather than charges and the GEE approach accounts for within-person correlation in repeated annual measurements and provides population-averaged estimates that are appropriate for health system planning.

Our findings principally provide detailed estimates of annual healthcare costs and their evolution over calendar time for this rare malignancy, thereby supplying empirically based cost inputs for future cost-effectiveness analyses, including simulation models evaluating alternative treatment and follow-up strategies.

## 5. Conclusions

In this regional cohort of patients with UTUC, annual direct healthcare costs decreased between 2019 and 2023. In subgroup analyses, systemic treatment in patients operated with ONU, female sex in patients operated with SU, and comorbidity in individuals subjected to ET were associated with increased healthcare costs. The estimates defined can be used in future cost-effectiveness studies to, for example, optimise the use of surgical modalities and systemic therapies.

## Figures and Tables

**Table 1:** Background characteristics of 278 patients diagnosed with upper tract urothelial carcinoma in Region Skåne, Sweden, 2019–2023.

Characteristics		Number	Percentage
**Age group**			
	<65	34	12
	65–74	93	34
	75–80	89	32
	81+	62	22
**Sex**			
	Male	165	59
	Female	113	41
**Smoking status**			
	Never smoker	60	22
	Former smoker	82	29
	Current smoker	71	26
	Missing information	65	23
**ASA score**			
	ASA 1	16	6
	ASA 2	105	38
	ASA 3–4	157	56
**Body mass index**			
	Normal	84	31
	Overweight	121	45
	Obese	66	24
**Functional single kidney**			
	No	256	92
	Yes	22	8
**History of bladder cancer**			
	No	209	75
	Yes	69	25
**Previous contralateral UTUC**			
	No	272	98
	Yes	6	2
**Tumour size**			
	Below 20 mm	48	17
	20 mm and higher	191	69
	Missing information	39	14
**Tumour site**			
	Renal pelvis	156	56
	Upper ureter	27	10
	Middle ureter	19	7
	Distal ureter	76	27
**Multiple tumours**			
	No	257	92
	More than one tumour	21	8
**Local invasion on CT**			
	No	175	63
	Yes	103	37
**MDT referral**			
	No	17	6
	Yes	261	94
**EAU risk category**			
	Low	22	8
	High	256	92
**FISH analysis (voided/selective) performed**			
	No	246	88
	Yes	32	12
**Selective urine cytology obtained**			
	No	213	76
	Yes	65	24
**Biopsy performed via TUR**			
	No	264	95
	Yes	14	5
**Clinical tumour stage**			
	Ta-T1, CIS, or Tx	165	59
	T2–T4	113	41
**Clinical nodal stage**			
	N0	239	86
	N+	39	14
**Clinical metastasis stage**			
	M0	259	93
	M1	19	7
**Treatment intent**			
	Curative	255	92
	Palliative	23	8
**Treatment type**			
	RANU	144	52
	ONU	45	16
	RADU/ODU	40	14
	Endourology	26	9
	Palliative systemic	10	4
	Best supportive care	13	5
**Perioperative lymphadenectomy**			
	No	200	72
	Yes	78	28
**Systemic regimens**			
	Not received	200	72
	Carboplatin–Gemcitabine	50	18
	Cisplatin–Gemcitabine/MVAC	16	6
	Pembrolizumab/Nivolumab	12	4
**Adjuvant upper tract instillation**			
	No	263	95
	Yes	15	5
**Pathological stage groups**			
	N/A	19	7
	Ta/Tx and G1	16	6
	Ta/Tx and G2	72	26
	Ta/CIS/T1/Tx and G3	76	28
	T2–T4 (any grade)	92	33
**Pathological nodal stage**			
	N0	63	23
	N1–N2	15	5
	Nx	200	72

ASA = American Society of Anaesthesiologists, UTUC = upper tract urothelial carcinoma, CT = computed tomography, MDT = multidisciplinary tumour board discussion, EAU = European Association of Urology risk stratification of UTUC, FISH = fluorescence in situ hybridisation, TUR = transuretral resection, CIS = carcinoma in situ, RANU = robot-assisted nephroureterectomy, RADU = robot-assisted distal ureterectomy, ODU = open distal ureterectomy, ONU = open nephroureterectomy, MVAC = Metothrexate Vinblastine Doxorubicin and Cisplatin, N/A = not applicable.

**Table 2:** GLM log link of healthcare costs with univariable and multivariable cost ratios.

Characteristics	Unadjusted Cost Ratio	Unadjusted *p*-Value	Unadjusted 95% CI	Adjusted Cost Ratio	Adjusted *p*-Value	Adjusted 95% CI	Bonferroni-Corrected *p*-Value
**Age group**							
<65	1.00	--	--	1.00	--	--	--
65–74	0.78	0.260	0.50–1.21	0.98	0.895	0.68–1.40	1
75–80	0.92	0.714	0.57–1.46	1.08	0.660	0.76–1.53	1
81+	0.82	0.390	0.52–1.29	1.02	0.918	0.71–1.47	1
**Sex**							
Male	1.00	--	--	1.00	--	--	--
Female	0.98	0.889	0.77–1.25	0.98	0.827	0.79–1.20	1
**Smoking status**							
Never smoker	1.00	--	--	1.00	--	--	--
Former smoker	0.90	0.474	0.67–1.21	1.00	0.989	0.77–1.31	1
Current smoker	0.65	0.004	0.48–0.87	0.80	0.116	0.61–1.06	1
Missing	0.85	0.416	0.57–1.26	0.87	0.379	0.65–1.18	1
**ASA score**							
ASA 1	1.00	--	--	1.00	--	--	--
ASA 2	1.19	0.462	0.75–1.87	1.19	0.404	0.79–1.79	1
ASA 3–4	1.39	0.136	0.90–2.13	1.29	0.241	0.84–1.98	1
**Body mass index**							
Normal	1.00	--	--	1.00	--	--	--
Overweight	0.97	0.824	0.74–1.27	0.99	0.935	0.79–1.24	1
Obese	0.98	0.874	0.74–1.29	1.02	0.902	0.80–1.29	1
**History of bladder cancer**							
No	1.00	--	--	1.00	--	--	--
Yes	1.08	0.603	0.81–1.44	1.26	0.115	0.95–1.69	1
**Clinical tumour stage**							
Ta–T1, CIS, Tx	1.00	--	--	1.00	--	--	--
T2–T4	1.32	0.016	1.05–1.65	1.26	0.095	0.96–1.65	1
**Clinical nodal stage**							
N0	1.00	--	--	1.00	--	--	--
N+	1.58	0.000	1.22–2.05	0.96	0.833	0.67–1.38	1
**Treatment type**							
RANU	1.00	--	--	1.00	--	--	--
ONU	1.00	0.993	0.75–1.34	0.77	0.089	0.57–1.04	1
RADU/ODU	0.76	0.060	0.58–1.01	0.78	0.096	0.58–1.04	1
Endourology	0.87	0.428	0.62–1.22	0.91	0.666	0.59–1.41	1
Palliative systemic	2.16	0.000	1.61–2.91	1.15	0.510	0.75–1.77	1
BSC/No treatment	0.98	0.893	0.69–1.37	1.03	0.900	0.67–1.57	1
**Any systemic treatment**							
No	1.00	--	--	1.00	--	--	--
Yes	1.65	0.000	1.30–2.09	1.52	0.005	1.13–2.04	0.1

Estimates are reported as exponentiated coefficients (cost ratios) from gamma family models with log link, weighted by inverse probability weights and clustered by patient’s id number. Reference categories are the omitted baseline levels shown with cost ratio 1.00. Abbreviations: GLM = generalised linear model, ASA = American Society of Anaesthesiologists, CIS = carcinoma in situ, RANU = robot-assisted nephroureterectomy, RADU = robot-assisted distal ureterectomy, ODU = open distal ureterectomy, ONU = open nephroureterectomy.

## Data Availability

The data that support the findings of this study are not publicly available due to privacy and ethical restrictions. Researchers who meet the criteria for access to confidential data may apply for data access through the Swedish National Board of Health and Welfare (https://www.socialstyrelsen.se) and the Swedish Ethical Review Authority (https://etikprovningsmyndigheten.se) accessed on 19 February 2025.

## Supplementary Materials

The following supporting information can be downloaded at: https://www.mdpi.com/article/10.3390/healthcare14121729/s1, Table S1. Distribution of patients with upper tract urothelial carcinoma in Skåne, Sweden by year of diagnosis and vital status; Table S2. Annual number of hospital days per calendar year (2019–2023) by year of diagnosis in patients with upper tract urothelial carcinoma in Skåne, Sweden; Table S3. Annual number of outpatient contacts per calendar year (2019–2023) by year of diagnosis in patients with upper tract urothelial carcinoma in Skåne, Sweden; Table S4. Treatment type by year of treatment (numbers (%)) in patients with upper tract urothelial carcinoma in Skåne, Sweden; Table S5. Exponentiated predicted mean annual costs by calendar year for patients with upper tract urothelial carcinoma in Region Skåne, Sweden with unadjusted and adjusted covariates; Table S6. Mean annual healthcare costs (2023 international dollar) and sample size by year of diagnosis and follow-up year among patients with upper tract urothelial carcinoma in Skåne, Sweden; Table S7. GLM log-link regression of healthcare costs with univariable and multivariable cost ratios for robot-assisted nephroureterectomy in patients with upper tract urothelial carcinoma, Skåne Sweden; Table S8. GLM log-link regression of healthcare costs with univariable and multivariable cost ratios for open nephroureterectomy in patients with upper tract urothelial carcinoma, Skåne Sweden; Table S9. GLM log-link regression of healthcare costs with univariable and multivariable cost ratios for robot-assisted distal ureterectomy or open distal ureterectomy in patients with upper tract urothelial carcinoma, Skåne Sweden; Table S10. GLM log-link regression of healthcare costs with univariable and multivariable cost ratios for endourologic treatment in patients with upper tract urothelial carcinoma, Skåne Sweden; Table S11. Exponentiated predicted mean annual costs by calendar year for patients with upper tract urothelial carcinoma in Region Skåne, Sweden undergoing robot-assisted nephroureterectomy with unadjusted and adjusted covariates; Table S12. Exponentiated predicted mean annual costs by calendar year for patients with upper tract urothelial carcinoma in Region Skåne, Sweden undergoing open nephroureterectomy with unadjusted and adjusted covariates; Table S13. Exponentiated predicted mean annual costs by calendar year for patients with upper tract urothelial carcinoma in Region Skåne, Sweden undergoing segmental ureterectomy with unadjusted and adjusted covariates; Table S14. Exponentiated predicted mean annual costs by calendar year for patients with upper tract urothelial carcinoma in Region Skåne, Sweden undergoing endourologic treatment with unadjusted and adjusted covariates.
