# Supplementary material for: Healthcare Resource Utilisation in Patients with Upper Tract Urothelial Carcinoma
Source: Healthcare (Basel). 2026 Jun 16;14(12):1729. doi: 10.3390/healthcare14121729 (PMC13299672; doi:10.3390/healthcare14121729)
Supplement: Supplementary file 1 [file healthcare-14-01729-s001.zip › healthcare-4309305-supplementary.pdf]

Supplementary materials:

Supplementary Table S1. Distribution of patients with upper tract urothelial carcinoma in Skåne, Sweden by year of diagnosis and vital status.

Year of diagnosis	Alive, n (%)	Dead, n (%)	Total, n (%)
2019	38 (70.4)	16 (29.6)	54 (19.4)
2020	39 (78.0)	11 (22.0)	50 (18.0)
2021	50 (89.3)	6 (10.7)	56 (20.1)
2022	47 (79.7)	12 (20.3)	59 (21.2)
2023	55 (93.2)	4 (6.8)	59 (21.2)
Total	229 (82.4)	49 (17.6)	278 (100)

Note: Vital status assessed at 31 December 2023

Supplementary Table S2. Annual number of hospital days per calendar year (2019–2023) by year of diagnosis in patients with upper tract urothelial carcinoma in Skåne, Sweden.

Year of diagnosis.	Days 2019	Days 2020	Days 2021	Days 2022	Days 2023
2019	14.1 (16.7) [43]	12.5 (19.1) [32]	15.7 (31.8) [16]	23.6 (28.6) [21]	15.1 (19.0) [19]
2020	–	9.5 (5.9) [44]	13.1 (15.6) [27]	16.0 (28.8) [21]	13.1 (17.7) [23]
2021	–	–	13.7 (13.7) [41]	12.2 (9.6) [31]	11.7 (9.8) [18]
2022	–	–	–	12.6 (11.4) [43]	11.9 (14.4) [31]
2023	–	–	–	–	11.9 (12.5) [46]

Values are mean (standard deviation) [numbers] of total inpatient days per patient-year.

Supplementary Table S3. Annual number of outpatient contacts per calendar year (2019–2023) by year of diagnosis in patients with upper tract urothelial carcinoma in Skåne, Sweden.

Year of diagnosis.	Contacts 2019	Contacts 2020	Contacts 2021	Contacts 2022	Contacts 2023
2019	14.7 (13.4) [54]	25.5 (29.5) [52]	20.5 (26.3) [49]	16.4 (10.2) [46]	15.9 (10.0) [39]
2020	–	13.7 (8.3) [48]	23.5 (26.4) [48]	19.5 (24.6) [45]	20.9 (23.4) [42]
2021	–	–	12.8 (10.6) [56]	17.6 (9.5) [55]	16.3 (11.2) [50]
2022	–	–	–	11.6 (9.1). [57]	15.5 (9.3) [50]
2023	–	–	–	–	11.8 (8.2). [58]

Values are mean number of contacts per year (standard deviation) [numbers].

Supplementary Table S4. Treatment type by year of treatment (numbers (%)) in patients with upper tract urothelial carcinoma in Skåne, Sweden.

Treatment type	2019	2020	2021	2022	2023	Total
RANU	26 (18.1)	29 (20.1)	29 (20.1)	24 (16.7)	36 (25.0)	144
ONU	11 (24.4)	6 (13.3)	13 (28.9)	7 (15.6)	8 (17.8)	45

RADU / ODU	12 (30.0)	7 (17.5)	7 (17.5)	11 (27.5)	3 (7.5)	40
Endourology	2 (7.7)	7 (26.9)	4 (15.4)	8 (30.8)	5 (19.2)	26
Palliative systemic	1 (10.0)	0 (0.0)	2 (20.0)	3 (30.0)	4 (40.0)	10
Best supportive care	2 (15.4)	1 (7.7)	1 (7.7)	6 (46.2)	3 (23.1)	13
Total	54 (19.4)	50 (18.0)	56 (20.1)	59 (21.2)	59 (21.2)	278

Pearson chi-square test for association between treatment categories and calendar year p=0.11, RANU = Robot Assisted Nephroureterectomy, ONU = Open Nephroureterectomy, RADU = Robot Assisted Distal Ureterectomy, ODU = Open Distal Ureterectomy.

Supplementary Table S5. Exponentiated predicted mean annual costs by calendar year for patients with upper tract urothelial carcinoma in Region Skåne, Sweden with unadjusted and adjusted covariates.

Year	Unadjusted				Adjusted			
	Margin	Std. Err.	P value	95% CI	Margin	Std. Err.	P value	95% CI
2019	36,536	4330	<0.001	28049–45022	36,870	4059	<0.001	28915–44825
2020	36,334	3445	<0.001	29582–43087	36,731	3167	<0.001	30524–42938
2021	34,633	3876	<0.001	27037–42230	34,625	3766	<0.001	27243–42006
2022	29,614	2480	<0.001	24753–34476	28,793	2367	<0.001	24153–33433
2023	30,380	2786	<0.001	24920–35839	30,004	2646	<0.001	24817–35191

Note: GLM Gamma Log-Link Models (Delta-Method Standard Errors adjusted for clustering on person). Adjusted for Age group, Sex, Smoking status, ASA score, Body Mass Index, History of bladder cancer, Clinical tumour stage, Clinical nodal stage, Treatment type and Any systemic treatment.

Supplementary Table S6. Mean annual healthcare costs (2023 international dollar) and sample size by year of diagnosis and follow-up year among patients with upper tract urothelial carcinoma in Skåne, Sweden.

Year of diagnosis\year	2019	2020	2021	2022	2023
2019	36,536 (54)	33,929 (53)	29,050 (49)	22,402 (47)	21,486 (39)
2020	—	38,936 (49)	32,615 (48)	29,728 (45)	38,431 (42)
2021	—	—	41,249 (56)	29,383 (55)	19,869 (50)
2022	—	—	—	35,489 (59)	33,004 (50)
2023	—	—	—	—	37,211 (59)

Supplementary Table S7. GLM log-link regression of healthcare costs with univariable and multivariable cost ratios for robot-assisted nephroureterectomy in patients with upper tract urothelial carcinoma, Skåne Sweden.

Characteristics	Unadjusted cost ratio	Unadjusted p value	Unadjusted 95% CI	Adjusted cost ratio	Adjusted p value	Adjusted 95% CI	Bonferroni corrected p value
Age group							
<65	1.00	--	--	1.00	--	--	
65–74	0.64	0.288	0.28–1.46	0.72	0.308	0.39–1.35	1

75–80	0.84	0.683	0.36–1.95	0.85	0.613	0.46–1.59	1
81+	0.69	0.383	0.30–1.59	0.79	0.481	0.41–1.52	1
Sex							
Male	1.00	--	--	1.00	--	--	
Female	0.91	0.635	0.63–1.32	0.94	0.672	0.71–1.25	1
Smoking status							
Never smoker	1.00	--	--	1.00	--	--	
Former smoker	0.91	0.730	0.53–1.55	0.97	0.884	0.67–1.41	1
Current smoker	0.61	0.069	0.36–1.04	0.71	0.098	0.48–1.06	1
Missing	0.90	0.766	0.45–1.79	0.87	0.488	0.58–1.30	1
ASA score							
ASA 1	1.00	--	--	1.00	--	--	
ASA 2	1.98	0.035	1.05–3.74	1.92	0.023	1.09–3.38	0.345
ASA 3–4	2.29	0.003	1.34–3.93	2.01	0.013	1.16–3.50	0.195
Body Mass Index							
Normal	1.00	--	--	1.00	--	--	
Overweight	0.99	0.957	0.64–1.52	1.04	0.840	0.73–1.47	1
Obese	1.08	0.683	0.74–1.58	1.18	0.237	0.90–1.57	1
History of bladder cancer							
No	1.00	--	--	1.00	--	--	
Yes	1.56	0.122	0.89–2.75	1.83	0.021	1.09–3.04	0.315
Clinical tumour stage							
Ta–T1, CIS, Tx	1.00	--	--	1.00	--	--	
T2–T4	1.22	0.258	0.86–1.72	1.22	0.287	0.84–1.78	1
Clinical nodal stage							
N0	1.00	--	--	1.00	--	--	
N+	1.96	0.005	1.23–3.12	1.45	0.242	0.78–2.71	1
Any systemic treatment							
No	1.00	--	--	1.00	--	--	
Yes	1.48	0.063	0.98–2.23	1.28	0.175	0.90–1.82	1

GLM = Generalised Linear Model, ASA = American Society of Anaesthesiologists, CIS = Carcinoma In Situ. Estimates are reported as exponentiated coefficients (cost ratios) from gamma family models with log link, weighted by inverse probability weights and clustered by patient's ID number. Reference categories are the omitted baseline levels shown with cost ratio 1.00.

Supplementary Table S8. GLM log-link regression of healthcare costs with univariable and multivariable cost ratios for open nephroureterectomy in patients with upper tract urothelial carcinoma, Skåne Sweden.

Characteristics	Unadjusted cost ratio	Unadjusted p value	Unadjusted 95% CI	Adjusted cost ratio	Adjusted p value	Adjusted 95% CI	Bonferroni corrected p value
Age group							
<65	1.00	--	--	1.00	--	--	
65–74	0.85	0.582	0.47–1.52	1.10	0.660	0.72–1.68	1
75–80	1.00	0.999	0.54–1.84	0.92	0.693	0.61–1.39	1
81+	0.79	0.656	0.28–2.23	0.79	0.503	0.39–1.58	1
Sex							
Male	1.00	--	--	1.00	--	--	
Female	0.95	0.861	0.57–1.60	1.26	0.300	0.81–1.94	1
Smoking status							
Never smoker	1.00	--	--	1.00	--	--	
Former smoker	0.95	0.830	0.58–1.55	1.41	0.163	0.87–2.29	1
Current smoker	0.53	0.005	0.34–0.83	0.65	0.118	0.38–1.12	1
Missing	1.09	0.826	0.52–2.29	1.50	0.092	0.94–2.40	1
ASA score (DCI) three groups							
ASA 1	1.00	--	--	1.00	--	--	
ASA 2	1.30	0.335	0.76–2.24	0.98	0.964	0.46–2.11	1
ASA 3–4	0.91	0.716	0.56–1.49	0.73	0.436	0.33–1.62	1
Body Mass Index							
Normal	1.00	--	--	1.00	--	--	
Overweight	1.32	0.326	0.76–2.31	1.03	0.883	0.67–1.59	1
Obese	0.90	0.682	0.54–1.50	1.02	0.935	0.63–1.65	1
History of bladder cancer							
No	1.00	--	--	1.00	--	--	
Yes	1.09	0.725	0.68–1.73	0.90	0.712	0.53–1.55	1
Clinical tumour stage							
Ta–T1, CIS, Tx	1.00	--	--	1.00	--	--	
T2–T4	1.17	0.565	0.68–2.02	0.61	0.012	0.41–0.90	0.18
Clinical nodal stage							
N0	1.00	--	--	1.00	--	--	
N+	1.12	0.614	0.72–1.74	0.91	0.699	0.55–1.49	1
Any systemic treatment							
No	1.00	--	--	1.00	--	--	

Yes	2.06	<0.001	1.43–2.97	2.38	<0.001*	1.56–3.62	<0.001

Note: *; Statistically significantly after Bonferroni correction. Estimates are reported as exponentiated coefficients (cost ratios) from gamma family models with log link, weighted by inverse probability weights and clustered by patient's ID number. Reference categories are the omitted baseline levels shown with cost ratio 1.00. Abbreviations: GLM = Generalised Linear Model; ASA = American Society of Anesthesiologists; CIS = Carcinoma in situ.

Supplementary Table S9**.** GLM log-link regression of healthcare costs with univariable and multivariable cost ratios for robot-assisted distal ureterectomy or open distal ureterectomy in patients with upper tract urothelial carcinoma, Skåne Sweden.

Characteristics	Unadjusted cost ratio	Unadjusted p value	Unadjusted 95% CI	Adjusted cost ratio	Adjusted p value	Adjusted 95% CI	Bonferroni corrected p value
Age group							
<65	1.00	--	--	1.00	--	--	
65–74	1.18	0.597	0.64–2.15	0.91	0.680	0.57–1.44	1
75–80	0.86	0.631	0.46–1.59	0.64	0.126	0.36–1.13	1
81+	1.82	0.071	0.95–3.48	1.97	0.005	1.23–3.15	0.075
Sex							
Male	1.00	--	--	1.00	--	--	
Female	1.46	0.073	0.97–2.22	1.82	<0.001*	1.30–2.55	<0.001
Smoking status							
Never smoker	1.00	--	--	1.00	--	--	
Former smoker	0.83	0.599	0.41–1.68	1.75	0.153	0.81–3.75	1
Current smoker	0.85	0.660	0.42–1.73	2.03	0.063	0.96–4.28	0.945
Missing	0.77	0.456	0.39–1.52	1.63	0.192	0.78–3.37	1
ASA score							
ASA 1	1.00	--	--	1.00	--	--	
ASA 2	0.75	0.388	0.39–1.45	0.93	0.803	0.50–1.70	1
ASA 3–4	0.95	0.898	0.45–2.01	0.83	0.633	0.39–1.77	1
Body Mass Index							
Normal	1.00	--	--	1.00	--	--	
Overweight	0.83	0.460	0.50–1.36	1.46	0.082	0.95–2.23	1
Obese	0.80	0.459	0.45–1.44	1.86	0.013	1.14–3.03	0.195
History of bladder cancer							
No	1.00	--	--	1.00	--	--	
Yes	0.85	0.449	0.56–1.29	1.18	0.348	0.84–1.66	1
Clinical tumour stage							
Ta–T1, CIS, Tx	1.00	--	--	1.00	--	--	
T2–T4	1.74	0.006	1.18–2.58	1.44	0.117	0.91–2.29	1

Clinical nodal stage							
N0	1.00	--	--	1.00	--	--	
N+	1.82	0.199	0.73–4.54	0.79	0.609	0.32–1.94	1
Any systemic treatment							
No	1.00	--	--	1.00	--	--	
Yes	1.66	0.065	0.97–2.85	1.55	0.037	1.03–2.34	0.555

Note: *; Statistically significantly after Bonferroni adjustment. Estimates are reported as exponentiated coefficients (cost ratios) from gamma family models with log link, weighted by inverse probability weights and clustered by patient's ID number. Reference categories are the omitted baseline levels shown with cost ratio 1.00. Abbreviations: GLM = Generalised linear model, ASA = American Society of Anaesthesiologists, CIS = Carcinoma In Situ

Supplementary Table S10. GLM log-link regression of healthcare costs with univariable and multivariable cost ratios for endourologic treatment in patients with upper tract urothelial carcinoma, Skåne Sweden.

Characteristics	Unadjusted cost ratio	Unadjusted p value	Unadjusted 95% CI	Adjusted cost ratio	Adjusted p value	Adjusted 95% CI	Bonferroni corrected p value
Age group							
<65	1.00	--	--	1.00	--	--	
65–74	2.25	0.009	1.22–4.14	1.02	0.961	0.47–2.20	1
75–80	2.35	0.002	1.38–4.01	1.42	0.128	0.90–2.24	1
81+	1.36	0.280	0.78–2.39	1.11	0.694	0.67–1.84	1
Sex							
Male	1.00	--	--	1.00	--	--	
Female	0.63	0.169	0.33–1.21	0.57	0.070	0.31–1.05	0.84
Smoking status							
Never smoker	1.00	--	--	1.00	--	--	
Former smoker	1.32	0.354	0.73–2.38	0.99	0.971	0.49–1.97	1
Current smoker	1.35	0.500	0.57–3.20	1.29	0.644	0.44–3.79	1
Missing	0.57	0.098	0.30–1.11	0.50	0.033	0.27–0.95	0.396
ASA score							
ASA 1	1.00	--	--	1.00	--	--	
ASA 2	3.15	<0.001	2.01–4.93	3.22	0.002*	1.55–6.71	0.024
ASA 3–4	6.10	<0.001	4.48–8.32	5.46	<0.001*	3.18–9.37	<0.001
Body Mass Index							
Normal	1.00	--	--	1.00	--	--	
Overweight	0.77	0.445	0.40–1.50	0.61	0.072	0.36–1.05	0.864
Obese	0.74	0.418	0.35–1.54	0.75	0.493	0.32–1.72	1
History of bladder cancer							
No	1.00	--	--	1.00	--	--	
Yes	1.27	0.516	0.62–2.57	0.95	0.880	0.49–1.84	1

Note: *; Statistically significantly after Bonferroni correction. Estimates are reported as exponentiated coefficients (cost ratios) from gamma family models with log link, weighted by inverse probability weights and clustered by patient's ID number. Reference categories are the omitted baseline levels shown with cost ratio 1.00. Abbreviations GLM = Generalised linear model, ASA = American Society of Anaesthesiologists

Supplementary Table S11. Exponentiated predicted mean annual costs by calendar year for patients with upper tract urothelial carcinoma in Region Skåne, Sweden undergoing robot-assisted nephroureterectomy with unadjusted and adjusted covariates.

Year	Unadjusted				Adjusted			
	Margin	Std. Err.	P value	95% CI	Margin	Std. Err.	P value	95% CI
2019	36,748	7523	<0.001	22003–51494	35,410	5533	<0.001	24565–46254
2020	39,018	5068	<0.001	29086–48951	38,965	4350	<0.001	30439–47491
2021	38,860	6164	<0.001	26779–50941	36,959	5074	<0.001	27014–46904
2022	30,150	3890	<0.001	22526–37775	29,062	3303	<0.001	22588–35536
2023	28,585	4173	<0.001	20407–36764	30,697	4277	<0.001	22315–39080

Note: GLM Gamma Log-Link Models (Delta-Method Standard Errors adjusted for clustering on person). Adjusted for Age group, Sex, Smoking status, ASA score, Body Mass Index, History of Bladder cancer, Clinical tumour stage, Clinical nodal stage, and any systemic treatment.

Supplementary Table S12. Exponentiated predicted mean annual costs by calendar year for patients with upper tract urothelial carcinoma in Region Skåne, Sweden undergoing open nephroureterectomy with unadjusted and adjusted covariates.

Year	Unadjusted				Adjusted			
	Margin	Std. Err.	P value	95% CI	Margin	Std. Err.	P value	95% CI
2019	33,529	4 490	<0.001	24 728–42 329	51,815	13 096	<0.001	26 147–77 483
2020	30,138	5 765	<0.001	18 837–41 439	39,011	11 245	0.001	16 971–61 052
2021	34,025	7 176	<0.001	19 959–48 091	36,314	8 118	<0.001	20 402–52 226
2022	31,171	6 157	<0.001	19 103–43 238	28,402	5 135	<0.001	18 337–38 468
2023	35,024	7 203	<0.001	20 906–49 142	30,950	5 855	<0.001	19 474–42 426

Note: GLM Gamma Log-Link Models (Delta-Method Standard Errors adjusted for clustering on person). Adjusted for Age group, Sex, Smoking status, ASA score, Body Mass Index, History of Bladder cancer, Clinical tumour stage, Clinical nodal stage, and any systemic treatment.

Supplementary Table S13. Exponentiated predicted mean annual costs by calendar year for patients with upper tract urothelial carcinoma in Region Skåne, Sweden undergoing segmental ureterectomy with unadjusted and adjusted covariates.

Year	Unadjusted				Adjusted			
	Margin	Std. Err.	P value	95% CI	Margin	Std. Err.	P value	95% CI
2019	33,007	6677	<0.001	19920–46093	46,428	17332	<0.001	12458–80397
2020	30,710	6044	<0.001	18863–42557	37,272	9513	<0.001	18627–55918
2021	25,411	6753	<0.001	12175–38646	24,391	5024	<0.001	14544–34238

2022	25,283	4449	<0.001	16564–34002	25,864	4489	<0.001	17066–34661
2023	19,784	4143	<0.001	11663–27904	16,610	2411	<0.001	11885–21334

Note: GEE Gamma Log-Link Models (Delta-Method Standard Errors adjusted for clustering on person). Adjusted for Age group, Sex, Smoking status, ASA score, Body Mass Index, History of bladder cancer, Clinical tumour stage, Clinical nodal stage, and Any systemic treatment.

Supplementary Table S14. Exponentiated predicted mean annual costs by calendar year for patients with upper tract urothelial carcinoma in Region Skåne, Sweden undergoing endourologic treatment with unadjusted and adjusted covariates.

Year	Unadjusted				Adjusted			
	Margin	Std. Err.	P value	95% CI	Margin	Std. Err.	P value	95% CI
2019	59,095	22344	0.008	15301–102889	87,545	45553	0.055	-1737–176828
2020	31,446	9090	0.001	13629–49263	29,302	7497	<0.001	14609–43996
2021	25,172	9592	0.009	6372–43973	20,349	5268	<0.001	10025–30674
2022	15,740	3405	<0.001	9067–22413	18,050	4528	<0.001	9174–26925
2023	38,326	7783	<0.001	23071–53581	37,731	6904	<0.001	24199–51263

Note: GLM Gamma Log-Link Models (Delta-Method Standard Errors adjusted for clustering on person). Adjusted for Age group, Sex, Smoking status, ASA score and Body Mass Index.
